# Coronavirus disease 2019 vaccination acceptance and associated factors among residents of Dire Dawa Administration, Eastern Ethiopia: a community-based cross-sectional study

**DOI:** 10.1186/s12879-024-09556-x

**Published:** 2024-07-11

**Authors:** Tafese Dejene Jidha, Endayen Deginet, Bereket Tefera, Demisew Amenu, Girma Beressa, Mickiale Hailu

**Affiliations:** 1 College of Medicine and Health Sciences, Dire Dawa University, Dire Dawa, Ethiopia; 2 Department of Obstetrics & Gynecology, Jimma University Medical Center, Jimma, Ethiopia; 3 Faculty of Public Health, Jimma University, Jimma, Ethiopia

**Keywords:** Coronavirus disease 19 vaccine acceptance, Associated factors, Residents, Dire Dawa Administration

## Abstract

**Background:**

Corona virus disease 2019 (COVID-19) is an extremely contagious viral infection caused by the severe acute respiratory syndrome coronavirus 2. Understanding the willingness of the community to receive the COVID-19 vaccine will help in the development and implementation of effective COVID-19 vaccination promotion programs. Therefore, we aimed to assess the level of COVID-19 vaccine acceptance and associated factors among residents of Dire Dawa Administration, Eastern Ethiopia.

**Methods:**

A community-based cross-sectional study was conducted among 840 residents in Dire Dawa Administration from May 18th to June 18th, 2021. Multistage cluster sampling followed by systematic random sampling was used to select respondents. A pretested interviewer-administered structured questionnaire was used to collect the data from respondents. Bivariable and multivariable logistic regression were used to identify predictors of COVID-19 vaccine acceptance. The odds ratio (OR), along with a 95% confidence interval (CI), was used to estimate the strength of the association.

**Results:**

Out of 840 respondents recruited, the proportion of COVID-19 vaccine acceptance was found to be 54.4% (457/840); (95% CI: 51.0%, 57.7%) Being male [AOR = 1.85, 95% CI: (1.35, 2.54), *P* < 0.001], not having a current habit of substance use [AOR = 2.38, 95% CI: (1.73, 3.26), *P* < 0.001], having a monthly income of less than 51.31 USD [AOR = 0.19, 95% CI: (0.04, 0.88), *P* = 0.001]; and not having a prior history of vaccination experience [AOR = 0.40, 95% CI: (0.29, 0.54), *P* < 0.001] were significantly associated with COVID-19 vaccine acceptance.

**Conclusion:**

This study reveals that the proportion of COVID-19 vaccine acceptance among residents of Dire Dawa Administration, Eastern Ethiopia, was 54.4%. Factors like being male and not having a current habit of substance use were positively associated, whereas having a monthly income of less than 51.31 USD and not having a prior history of vaccination experience were negatively associated with COVID-19 vaccine acceptance. Health information dissemination and economic empowerment are crucial to improving COVID-19 vaccine acceptance among the community. This study provides valuable data for policymakers to plan early vaccination programs and tackle the challenges identified in the study.

**Supplementary Information:**

The online version contains supplementary material available at 10.1186/s12879-024-09556-x.

## Background

The novel coronavirus disease 2019 (COVID-19) is caused by the severe acute respiratory syndrome coronavirus 2 (SARS-CoV-2). A SARS-CoV-2 infection has been associated with a wide spectrum of illnesses that range from asymptomatic, mild to severe, or fatal [1]. To curb the spread of the virus and mitigate its health effects, different control measures have been implemented, such as wearing a face mask in public, social distancing, avoiding large gatherings, minimizing touching of the eyes, nose, and mouth, and washing hands often with soap and water [2].

Although such control measures have helped to curb the spread of the virus, the resurgence of the virus has been reported as people are not adhering to control measures, and economies and societies have reopened. Hence, there is an urgent need for long-term preventive measures [3]. The Royal Society and the British Academy which stated that a community-level vaccine coverage of 80+% will be required to protect the community from infection, dependent on the vaccine efficacy and duration of protection. However, it might be difficult to achieve these levels of vaccination coverage because of well-documented misinformation about vaccines across the world [4].

Furthermore, multiple factors influence vaccine acceptance, including safety concerns, the effectiveness of vaccines, perceived benefits of the vaccine, the susceptibility of individuals to COVID-19 infection, and the compatibility of vaccination with religious beliefs [5–7]. There has been widely circulating false information about the COVID-19 pandemic on social media. These include that the virus is linked with 5G mobile networks that the viral pandemic is a bio-weapon, and that vaccine trial participants have died after taking the vaccine. Such misleading data increases people’s anxieties, sows uncertainty about novel vaccinations, and restricts public acceptance of COVID-19 vaccinations [8–10].

Previous studies report the following factors as being significantly associated with COVID-19 vaccine acceptance in low-income countries including Ethiopia: Age, residence, gender, educational attainment, employment status, marital status, religion, profession, mass media, substance use, receiving any vaccination during childhood, and family members [11–16].

A COVID-19 vaccine has been hoped as the ultimate solution needed to end the COVID-19 pandemic. Studies pointed out that the health benefits of approved COVID-19 vaccines are undermined by hesitancy from populations to be vaccinated [17]. Community-level vaccine coverage of 80–90% is required to protect the community from the infection. Yet surveys emerging from diverse contexts illustrate widespread hesitance towards COVID-19 vaccines, with large differences between and within countries [18].

Currently, in Ethiopia, the Ministry of Health (MOH) has started providing AstraZeneca vaccines on March 6, 2021, with priority for front-line health workers and support staff, the elderly with underlying conditions, and other high-risk groups. Nevertheless, there was a paucity of information about the acceptance of a potential COVID-19 vaccine and associated factors among residents in Dire Dawa Administration, Eastern Ethiopia. Therefore, in this study, we sought to assess the acceptance of a potential COVID-19 vaccine and identify factors that influence its acceptance among the adult population in Dire Dawa, Eastern Ethiopia. The findings of this study would help public health experts at both regional and federal levels develop health messages on these factors to improve COVID-19 vaccine acceptance.

## Methods and materials

### Study setting

Dire Dawa Administration is located 515 km away from Addis Ababa to the east. There are 9 urban kebeles and 38 rural kebeles in the Dire Dawa Administration (Kebele is the smallest administrative unit in the district of Ethiopia). It has attained complete access to primary health care. In terms of health care facilities, the administration has two governmental and four private hospitals, eight health centers, five high-level clinics, and twelve medium-sized clinics. The metro area population of the Dire Dawa administration in 2021 is 526,000, of which the adult population (age ≥ 18 years) accounts for 322,966. The estimated adult populations (≥ 18 years) who reside in urban and rural kebeles were 220,360 (68.2%) and 102,606 (31.8%), respectively. (Dire Dawa Administration Annual Report, 2020/21).

### Study design and subjects

A community-based cross-sectional study was conducted in the Dire Dawa Administration from May 18th to June 18th, 2021. All volunteer permanent residents (age ≥ 18 years) of the Dire Dawa Administration were the source population. All randomly selected volunteer permanent residents (age ≥ 18 years) of the Dire Dawa Administration were the study population. Residents with the following conditions were excluded from the study: individuals who were seriously ill during data collection, individuals having psychiatric problems, individuals who hadn’t resided in the study setting for at least 6 months, and individuals who had already been vaccinated.

### Sample size determination and sampling techniques

Sample size was computed using EPi info, considering COVID-19 acceptance, population size (since the estimated number of adults was assumed to be > 10,000), expected frequency = 53.6, [19] confidence limits = 5%, confidence level = 95%, DE = 2, and number of clusters (9 urban kebeles and 38 rural kebeles) = 47, which yielded a sample size of 799. Considering a 5% non-response rate, the final sample size was 840. Of the 9 urban kebeles and 38 rural peasants in Dire Dawa, 3 urban kebeles and 11 rural kebeles were selected using a multistage cluster sampling method. Initially, the study clusters were stratified into urban and rural areas. Then it was stratified into randomly selected clusters. Then, the sample size was proportionally allocated to each cluster, and residents were selected using systematic random sampling, with a K value calculated for each cluster. From the specific households selected, only one randomly selected eligible individual was interviewed. If a respondent was absent during the interview, the house of the absent respondent was revisited the next day.

### Data collection tools and methods

Pretested interviewer-administered structured questionnaires were used to collect the data. The tool was adapted from previously published studies, and all the co-variates were included from previous studies [11–13, 20]. The questionnaires were comprised of different sections: socio-demographic characteristics, general health status, personal and close experience with COVID-19 infection, previous vaccination experiences, and attitude toward COVID-19 vaccination. Data were collected by 14 trained registered nurses and four supervisors. The data collectors filled out the interviewer-administered surveys on pencil and paper. Study participants were invited to participate by knocking their doors but safety precautions toward COVID-19 prevention were taken during the data collection process. All data collectors and supervisors strictly adhered to the WHO and national standards for COVID-19 prevention protocols. The data collectors wore face masks, gloved their hands, maintained distance, and sanitized their hands between each interview.

### Data quality control

The questionnaire was prepared in English and translated into ‘Amharic’ and ‘Afan Oromo’ local languages. Then, it was back-translated into English by another fluent speaker to ensure consistency. To maintain the quality of the data, training was given to data collectors and supervisors on the objectives of the study, data collection instruments, and principles of research ethics before the data collection process. The questionnaire was pretested on 5% of the total sample size of participants (42 respondents) in the non-selected setting, and then necessary amendments were made. Each filled-out questionnaire was checked for completeness onsite by the supervisors, and necessary feedback was offered to the data collectors. Close supervision was conducted by investigators.

### Study variables and operational definitions

Family income was classified based on the African Development Bank definition [20].

To assess the level of COVID-19 vaccine acceptance, a yes-or-no question was asked: will you get vaccinated if the COVID-19 vaccine is available? It scored 0 and 1 (0 = no, 1 = yes).

There were nine questions to assess personal or close COVID-19 exposure, and each was scored between 0 and 1 (0 = no, 1 = yes). Those who scored less than the mean (less than 4.5 points) were assigned to have a lower COVID-19 exposure risk, whereas those who answered greater than or equal to 4.5 were assigned to have a higher COVID-19 exposure risk.

There were six questions to assess the attitude toward COVID-19 vaccine acceptance. The score of the attitude was based on a five-point Likert scale, on which a score of one to five was given, from strongly disagreeing to strongly agreeing. The total score was calculated by summing the raw scores of the six questions, with the composite score ranging between 6 and 30, with an overall greater score indicating more positive attitudes towards the COVID-19 vaccine.

### Data processing and analysis

Data were coded, checked, and entered into Epidata version 4.6; then, it was exported to SPSS version 26 for data analysis. Frequencies, percentages, median, and interquartile range (IQR) were used to summarize the study subjects. Concerning attitudes towards COVID-19 vaccine acceptance, a reliability analysis was conducted to see the interitem correlation, and Cronbach’s alpha was found to be 0.89. A cumulative mean and individual item mean were calculated, and a one-sample t-test was run to examine the participant’s attitude. The normality distribution of the data was assessed using the Kolmogorov-Smirnov test at a *P*-value of 0.05. Multi-collinearity was also checked to see the correlation between each independent variable, and the highest collinearity among all independent variables was 0.19, which was below the cut-off value. It showed that there were no interactions among independent variables.

Bivariable and multivariable logistic regression analyses were used to identify factors affecting COVID-19 vaccine acceptance. Variables with a *p*-value of < 0.25 in the bivariable regression analyses were included in the multivariable logistic regression analysis to adjust for all potential confounding effects [21–25]. Covariates included the following: sex of the respondents, marital status, occupation, family monthly income, the current habit of substance use, personal or close COVID-19 exposure, and previous other vaccination experiences. The odds ratio (OR), along with a 95% confidence interval (CI), was used to estimate the strength of the association between predictors and COVID-19 vaccine acceptance. Variables with a *p*-value of < 0.05 were considered statistically significant. The Hosmer and Lemeshow tests were used to check model fitness at a *P*-value of > 0.05.

### Ethical considerations

The research protocol was approved by the Research Ethics Review Committee of Dire Dawa University. A permission letter was obtained from the Dire Dawa Administration. A permission letter for data collection was also obtained from the respective kebele administrators. The method was conducted in accordance with the relevant tenets of the Helsinki Declaration. The Strengthening the Reporting of Observational Studies in Epidemiology (STROBE) statement guideline was used for reporting observational studies. The principles of Helsinki Declaration (2013) were sticked to keep the privacy, confidentiality and safety of the study participants [26]. Informed consent was obtained from each participant. Participants were informed that they had the right to withdraw from the study at any point during data collection. The privacy of participants was maintained by interviewing in isolated areas. Confidentiality of the data was assured by the anonymity of questionnaires, and they were kept in a locked cabinet. Participants were interviewed following the COVID-19 prevention protocol. Face masks were given to data collectors and study participants before the interview took place. At the end of the interview, the data collectors shared health information about COVID-19 prevention and vaccination to the study subjects.

## Results

### Socio-demographic and economic characteristics of the study participants

A total of 840 respondents participated in this study, with a response rate of 100%. The median age of respondents was 30 years old, varying from 18 to 72 years, with an IQR of 25–35 years. Of the total participants, the highest proportion, 766 (91.2%), were under 45 years old. 428 (51%) were females, 495 (59.2%) were married, while 390 (46.4%) were government employed. The highest proportion of participants resided in urban setting 548 (65.2%), and attained college and above, 414 (49.3%). Of the respondents, 498 (59.2%) of the participants belongs to middle family income, who had a monthly income of between 51.41 and 513.75. (Table 1)

**Table 1:** Socio-demographic and economic characteristics, health related factors, COVID-19 exposure experience and previous vaccination experience of residents in dire Dawa Administration, Eastern Ethiopia, 2021 (*N* = 840)

Variables	Frequency	Percent
Age (year)		
18–25 26–35 36–45 45 and above	198 383 185 74	23.6 45.6 22.0 8.8
Gender		
Male Female	412 428	49.0 51.0
Marital status		
Married Not married	495 345	59.2 40.8
Ethnicity		
Oromo Somali Amhara Tigre Others	313 100 331 44 52	37.3 11.9 39.4 5.2 6.2
Religion		
Muslim Orthodox Protestant Catholic Others	269 429 118 16 8	32.0 51.1 14.0 1.9 1.0
Occupation		
Student Government employee Private worker Merchant Unemployed Others	110 390 172 60 48 60	13.1 46.4 20.5 7.1 5.7 7.1
Education		
No formal education Primary school Secondary school College and above	46 172 208 414	5.5 20.5 24.8 49.3
Residence		
Urban Rural	548 292	65.2 34.8
Family monthly income		
<51.31 USD 51.31-513.75 USD ≥513.75 USD	328 498 14	39.1 59.2 1.7
Current alcohol drinker		
Yes No	209 631	24.9 75.1
Current cigarette smoker		
Yes No	130 710	15.5 84.5
Current khat chewer (plant based natural stimulant)		
Yes No	269 571	32.0 68.0
**Self-reported medical illnesses**		
Diabetes Mellitus		
Yes No	24 816	2.9 97.1
Hypertension		
Yes No	28 812	3.3 96.7
Chronic renal disease		
Yes No	10 830	1.2 98.8
Chronic respiratory disease		
Yes No	6 834	0.7 99.3
Heart disease		
Yes No	4 836	0.5 99.5
Cancer		
Yes No	2 838	0.2 99.8
Infected with COVID-19		
Yes No	50 790	6.0 94.0
Hospitalized due to COVID-19 infection		
Yes No	20 820	2.4 97.6
Family or close friend infected with COVID-19		
Yes No	203 637	24.2 75.8
Someone I knew infected with COVID-19		
Yes No	354 486	42.1 57.8
Exposed to someone infected with COVID-19		
Yes No	282 558	33.6 66.4
Treating patients infected with COVID-19		
Yes No	92 748	11.0 89.0
Taking care of someone infected with COVID-19		
Yes No	112 728	13.3 86.7
Have a family member or close friend died of COVID-19		
Yes No	133 707	15.8 84.2
Someone I knew died of COVID-19		
Yes No	518 322	61.7 38.3
Hepatitis B vaccination		
Yes No	232 608	27.6 72.4
Vaccinated for Tetanus		
Yes No	395 445	47.0 53.0
Vaccinated for Meningococcus		
Yes No	275 565	32.7 67.3

### Health-related factors of the study participants

The highest proportion, 725(86%) of the respondents had no known chronic medical illnesses, while very few respondents had reported of having diabetes mellitus (2.9%), hypertension (3.3%), chronic renal disease (1.2%), chronic respiratory disease (0.7%), heart disease (0.5%), cancer (0.2%) and HIV/AIDS (4.5%). Two hundred nine (24.9%) of the respondents reported they drank alcohol, while 130 (15.5%) and 269 (32.0%) reported current cigarette smoking and khat chewing respectively. (Table 1).

### COVID-19 exposure experience of the study participants

Among the study participants, 50 (6.0%) of the respondents had been tested positive for COVID-19, while 20 (2.4%) of the respondents reported that they were hospitalized because of COVID-19, and 203 (24.2%) of the respondents reported that they had family or close friends infected with COVID-19. Similarly, 354 (42.1%) of the respondents reported that they had known someone infected with COVID-19. Besides these, 282(33.6%) of the respondents reported that they were exposed to someone infected with COVID-19. Furthermore, 133 (15.8%) of the respondents reported that they had family or close friends who died of COVID-19, in addition 518 (61.7%) of the respondents reported that they knew someone who died of COVID-19 (Table 1).

### Previous vaccination experience among the study participants

Concerning previous vaccination experiences, 232(27.6%) of the respondents had a history of hepatitis B vaccination, 395(47.0%) and 275 (32.7%) of the respondents had a history of vaccination against tetanus and meningococcus (Table 1).

### Proportion of COVID-19 vaccine acceptance among the study participants

The proportion of COVID-19 vaccine acceptance was found to be 54.4%; (95% CI: (51, 57.7), whereas those who didn’t accept the COVID-19 vaccine were found to be 45.6%; (95% CI: 42.3, 49).

### Attitude toward Covid-19 vaccine acceptance

The calculated cumulative mean score showed that participants were not in favor of the presented items, with a mean of 2.96 at 95% CI: (-0.34, -0.17) (t (839) =-1.089, *p*-value < 0.001). Specifically, participants were not in favor of the vaccine in preventing COVID-19 (t (839) = -5.985, *p*-value < 0.001). Similarly, participants were not in favor of vaccination effectiveness (t (839) =-4.307, p < 0.001). Participants’ attitudes were also assessed with the question “If I get the vaccine, I will be less likely to get COVID-19,” and they were found to be negative (t (839) =-3.316, p 0.001). We also assessed if the COVID-19 vaccine protects the community from COVID-19 infection and found that the participants do not perceive it protects community health (t(839) =-5.33, *p*-value < 0.001). Participants’ attitudes toward vaccine safety were also assessed, and they revealed that they do not favor vaccine safety (t(839) =-3.599, *p*-value < 0.001). However, participants were not in favor of the “COVID-19 vaccine being easily available’ (t (839) = 41.86, *p*-value < 0.001) (Table 2).

**Table 2:** Attitude towards Covid-19 vaccine acceptance among residents of dire Dawa administration, Eastern Ethiopia, 2021 (*N* = 840)

Items	Test Value = 3				
	t	df	P	Mean	Mean Difference with 95% CI
COVID-19 vaccines will work in preventing the disease	-5.985	839	< 0.001	2.74	-0.258 (-0.34, -0.17)
Vaccines will be effective in preventing COVID-19	-4.307	839	< 0.001	2.82	-0.18 (-0.26, -0.1)
If I get the vaccines, I will be less likely to get COVID-19	-3.316	839	< 0.001	2.86	-0.137 (-0.22, -0.06)
COVID-19 vaccines protect the health of my community	-5.331	839	< 0.001	2.78	-0.219 (-0.3, -0.14)
COVID-19 vaccine is safe	-3.599	839	< 0.001	2.86	-0.14 (-0.22, -0.06)
COVID-19 vaccine is easily available	41.861	839	< 0.001	4.15	1.148 (1.09, 1.2)
Attitude Cumulative Mean	-1.089	839	< 0.001	2.96	-0.036 (-0.099, 0.029)

### Factors associated with COVID-19 vaccine acceptance

Respondents who were males [AOR = 1.85, 95% CI: (1.35, 2.54), *P* < 0.001] and who did not have a current habit of substance use [AOR = 2.38, 95% CI: (1.73, 3.26), *P* < 0.001] were more likely to get vaccinated. On the other hand, respondents having a monthly income of less than 51.31 USD [AOR = 0.19, 95% CI: (0.04, 0.88), *P* = 0.001]; and not having a prior history of vaccination experience were less likely to get vaccinated [AOR = 0.40, 95% CI: (0.29, 0.54), *P* < 0.001] (Table 3).

**Table 3:** Factors associated with COVID-19 vaccine acceptance among residents of dire Dawa administration, Eastern Ethiopia; 2021(*n* = 840)

Variables		COVID-19 vaccine acceptance		COR (95% CI)	AOR (95% CI)	*P*- value
		Yes (%)	No (%)			
Age of respondents	Age < 45 years age ≥ 45 years	413(49.2) 44(5.2)	353(42.0) 30(3.6)	1.254 (0.77, 2.04) Ref.	0.80 (0.49, 1.30) Ref.	0.361
Sex of respondents	Male Female	240(28.6) 217(25.8)	172(20.5) 211(25.1)	0.737(0.56, 0.97) Ref.	1.85 (1.35, 2.54) Ref.	< 0.001**
Marital status	Not married Married	166(19.8) 287(34.2)	175(20.8) 208(24.8)	0.687(0.52, 0.91) Ref.	0.75 (0.55, 1.01) Ref.	0.057
Educational status	Not attended formal education Attended formal education	26(3.1) 431(51.3)	20(2.4) 363(43.2)	0.913(0.50, 1.66) Ref.	1.10 (0.60, 1.99) Ref.	0.767
Occupation	Unemployed Employed	18(2.1) 439(52.3)	30(3.6) 353(42.0)	2.073(1.14, 3.78) Ref.	0.54 (0.29, 1.03) Ref.	0.620
Family monthly income	< 51.31 USD 51.31-513.75 USD ≥ 513.75 USD	128(15.2) 317(37.7) 12(1.4)	200(23.8) 181(21.5) 2(0.2)	2.768(2.10, 3.65) 0.58 (0.2, 1.5) Ref.	0.19 (0.04, 0.88) 0.43 (0.10, 2.02) Ref.	0.001** 0.288
Place of residence	Rural Urban	291(34.6) 166(19.8)	257(30.6) 126(15.0)	1.164(0.87, 1.55) Ref.	0.86 (0.65, 1.14) Ref.	0.299
Current substance use	No Yes	314(37.4) 143(17.0)	200(23.8) 183(21.8)	2.01(0.38, 0.66) Ref.	2.38 (1.73, 3.26) Ref.	< 0.001**
Medical illness	No Yes	407(48.5) 50(6.0)	331(39.4) 52(6.2)	0.782(0.52, 1.18) Ref.	1.30 (0.85, 1.94) Ref.	0.245
Personal or close friend COVID-19 exposure	No experience Has experience	118(14.0) 339(40.4)	148(17.6) 235(28.0)	0.553(1.35, 2.43) Ref.	0.77 (0.56, 1.06) Ref.	0.114
Previous vaccination history	No history Has history	138(16.4) 319(38.0)	199(23.7) 184(21.9)	0.4(1.88, 3.32) Ref.	0.40 (0.30, 0.54) Ref.	< 0.001**

Ref. – Reference; COR, Crude Odds ratio; AOR, Adjusted Odds Ratio; CI, Confidence interval

** - significant in multivariable logistic regression analysis

## Discussion

This study reveals that the proportion of COVID-19 vaccine acceptance among residents of Dire Dawa Administration, Eastern Ethiopia, was 54.4%. Being male, having a lower income in the family, not having a current habit of substance use, and not having previous vaccination experience were factors associated with COVID-19 vaccine acceptance.

The level of COVID-19 vaccine acceptance was found to be 54.4% in this study, which was higher than studies conducted in other parts of Ethiopia like among pregnant women who came for antenatal care service in the selected public health facilities Southwest Ethiopia (31.3%), among school teachers in Gonder city, Northwest Ethiopia (40.8%), among health professionals in Debre Tabor Comprehensive Specialized Hospital, North Central Ethiopia (42.3%), among community in Sodo town, Wolaita zone, southern Ethiopia (45.5%), and among health care workers in public hospitals in Kaffa zone and Bench-Sheko zone, Southwest Ethiopia (48.4%) [14–16, 27, 28]. 

But, it was lower than a study conducted among community in Gurage zone in Southern Ethiopia (62.6%), among university students in Northeast Ethiopia (69.3%), online survey conducted among health workers in Ethiopia (72.2%), among pregnant women attending antenatal care clinics, Southwest Ethiopia (70.7%), among patients with chronic disease in Northeastern Ethiopia (59.4%), among residents in Addis Ababa (80.93%) [29–34]. While, the findings of this study 54.4%; (95% CI: (51.0%, 57.7%), is actually comparable to the findings from the SRMA (56.02%) [35]. 

However, the current study’s findings was higher than of studies conducted in Cameroon (15.4%), Democratic Republic of Congo (25.3%), Uganda (37.3%), Nigeria (40.5%), and Ghana (39.3%) [36–40]. Additionally, the current study’s finding is higher than a systemic review and meta-analysis carried out in Africa (48.93%) [41].

Compared to other countries already mentioned, the higher rate of vaccine acceptance in Ethiopia could be attributed to the country’s proactive efforts in scaling up vaccination campaigns, deploying a large number of vaccinators, and ensuring the availability of a significant number of vaccine doses to reach a broader population base. Additionally, Ethiopia has received substantial support from international organizations like the World Bank, which has provided financial assistance to enhance the country’s vaccination efforts, particularly in rural areas and communities affected by conflicts. Nevertheless, our finding (54.4%.) was lower than the studies done in other countries like India (79%), Malaysian (83.3%) China (91.3%) and USA (75%) [42–45]. The possible explanation could be, in contrast, Malaysia and the United States have higher vaccine coverage rates than Ethiopia due to a combination of factors, including higher vaccine acceptance rates, better healthcare infrastructure and resources, and more effective vaccine communication and outreach strategies.

In our current study, we found that males were 1.85 times more likely to get vaccinated against the COVID-19 vaccine as compared to female respondents. This study agreed with studies conducted in Sodo Town [16]. Similarly, the present study’s findings agreed with studies carried out in Kuwait, [13] Philadelphia, [46] and the USA [47]. This may be due to the reason that women might show varying levels of COVID-19 vaccine hesitancy for several reasons. Some women express concerns about how COVID-19 vaccines may affect conception, pregnancy, and breastfeeding, influenced by warnings issued by health authorities in some countries. Additionally, women may rely more on online social networks for health information, where misinformation about COVID-19 and vaccines spreads, contributing to vaccine hesitancy. Nevertheless, it disagreed with studies conducted in Bangladesh [48], in which females were more willing to receive COVID-19 vaccines compared to their counterparts.

The current study also revealed that respondents with lower family monthly incomes were 81% less likely to get vaccinated than those with higher monthly incomes. This finding agreed with the study done in Bangladesh [48], Japan, [49] England, [50] and the USA [51]. A global survey regarding COVID-19 vaccine acceptance in 19 countries with 13,426 respondents also found less vaccine acceptance among participants with low income [52]. Concerns about vaccine cost and availability were the most common reasons given for vaccine hesitancy among low-income respondents. Policy initiatives focused on access barriers and vaccine costs will need to be coupled with communication efforts to make sure that those who need them are aware of the options available to them.

In this study, it was found that respondents who had no current habit of substance use (like cigarette smoking, alcohol, or khat) were 2.38 times more likely to get vaccinated compared to those with a current habit of substance use, which indicated that current substance users were more vaccine-hesitant than current substance non-users. A survey conducted among UK adults also found that current cigarette smokers held a negative attitude towards the COVID-19 vaccine, and were more likely to be unwilling to vaccinate against it, compared with never and former smokers [53].

The current substance users reported the greatest level of mistrust in the benefits of vaccines, worries about vaccine safety, fear of vaccines reverting to diseases, and preference for natural immunity. There is potential to address the vaccination intention gap through increasing awareness of the benefits of vaccination and correcting any potential misconceptions about the vaccine. Similarly, the current study’s finding agreed with a longitudinal study conducted in the USA, in which the use of cigarettes, marijuana, and heavy drinking alcohol was not associated with COVID-19 vaccine hesitancy [54].

In our study, respondents who had no prior vaccination experience like hepatitis B virus vaccine, meningococcal vaccine, or tetanus toxoid vaccine were 60% less likely to get vaccinated against COVID-19 than those who had prior vaccination experience, which showed that respondents with previous vaccination experience were more willing to vaccinate against COVID-19 than those who had no prior vaccination experience. This finding agreed with the study carried out in Kuwait, [13] Slovenia, [55] and Israel, [56] which showed participants who had previously received influenza vaccine were more likely to accept the COVID-19 vaccine compared to those who had not been vaccinated.

This study provides valuable data for policymakers to plan vaccination programs and tackle the challenges identified in the study. So based on this finding the following recommendations can be made:

- Develop targeted communication strategies that address the concerns and misconceptions about COVID-19 vaccines among populations with lower vaccine acceptance rates, such as those with lower monthly incomes and those without a prior history of vaccination experience.
- Improve access to COVID-19 vaccines in communities with lower vaccine acceptance rates, such as low-income communities, by providing free or low-cost vaccines and increasing the availability of vaccines in community health centers and other accessible locations.
- Leverage the influence of trusted community leaders, such as religious leaders, community health workers, and local celebrities, to promote COVID-19 vaccine acceptance and address concerns and misconceptions about vaccines.
- Address substance use and vaccination: Address the relationship between substance use and vaccination by providing education and resources to individuals with substance use disorders about the importance of vaccination and the potential risks of vaccine refusal.
- Monitor vaccine acceptance rates over time and adjust communication strategies and outreach efforts as needed to address changes in vaccine acceptance rates and emerging concerns and misconceptions about vaccines.
- Address social determinants of health, such as poverty and lack of access to healthcare, that contribute to lower COVID-19 vaccination rates in disadvantaged populations.
- Encourage vaccination among healthcare workers: Encourage vaccination among healthcare workers, who are often seen as trusted sources of information about vaccines, to promote vaccination among patients and the broader community.
- Continue research on vaccine acceptance: Continue research on vaccine acceptance and the factors that influence vaccine acceptance to inform the development of targeted communication strategies and outreach efforts.

### Strength and limitations of the study

This study was conducted in a community-based setting, encompassing both urban and rural residents and a large sample size, suggesting its potential generalizability to similar study settings. Even though, participant’s right were assured while interviewing, assessing coronavirus vaccination acceptance during the pandemic might introduce social desirability bias. To minimize these biases, the study implemented random selection of study participants.

## Conclusion

This study reveals that the proportion of COVID-19 vaccine acceptance among residents of Dire Dawa Administration, Eastern Ethiopia, was 54.4%. This finding was found relatively higher than the studies reported from some African countries Factors like being male and not having a current habit of substance use were positively associated, whereas having a monthly income of less than 51.31 USD and not having a prior history of vaccination experience were negatively associated with COVID-19 vaccine acceptance. However, the multidimensional national efforts should be sustained to leverage the vaccine acceptance rate. Considerations to improve adequate health information and access to vaccination service tailored to participant’s demographics, economic assistance, SBCC interventions directed at substance use might improve coronavirus vaccination acceptance.

### Electronic supplementary material

Below is the link to the electronic supplementary material.

Supplementary Material 1

## Data Availability

Data related to this manuscript is available on the hand of corresponding author and will be obtained under a reasonable request.

## Abbreviations

AOR: Adjusted Odd Ratio
BSc: Bachelors of Science
CI: Confidence interval
COR: Crude Odds ratio
COVID: 19-Coronavirus Disease 19
FDA: Food and Drug Administration
IQR: Interquartile range
WHO: World Health Organization
SARS: CoV-2-Severe Acute Respiratory Syndrome Coronavirus 2
SPSS: Statistical Package for the Social Sciences
UK: United Kingdom
USA: United States of America
USD: United States Dollars
